# A novel ensemble learning method for *de novo *computational identification of DNA binding sites

**DOI:** 10.1186/1471-2105-8-249

**Published:** 2007-07-12

**Authors:** Arijit Chakravarty, Jonathan M Carlson, Radhika S Khetani, Robert H Gross

**Affiliations:** 1 Department of Cancer Pharmacology, Millennium Pharmaceuticals Inc., Cambridge, MA, USA; 2 Department of Computer Science and Engineering, University of Washington, Seattle, WA, USA; 3 Department of Biological Sciences, Dartmouth College, Hanover, NH, USA

## Abstract

**Background:**

Despite the diversity of motif representations and search algorithms, the *de novo *computational identification of transcription factor binding sites remains constrained by the limited accuracy of existing algorithms and the need for user-specified input parameters that describe the motif being sought.

**Results:**

We present a novel ensemble learning method, SCOPE, that is based on the assumption that transcription factor binding sites belong to one of three broad classes of motifs: non-degenerate, degenerate and gapped motifs. SCOPE employs a unified scoring metric to combine the results from three motif finding algorithms each aimed at the discovery of one of these classes of motifs. We found that SCOPE's performance on 78 experimentally characterized regulons from four species was a substantial and statistically significant improvement over that of its component algorithms. SCOPE outperformed a broad range of existing motif discovery algorithms on the same dataset by a statistically significant margin.

**Conclusion:**

SCOPE demonstrates that combining multiple, focused motif discovery algorithms can provide a significant gain in performance. By building on components that efficiently search for motifs without user-defined parameters, SCOPE requires as input only a set of upstream sequences and a species designation, making it a practical choice for non-expert users. A user-friendly web interface, Java source code and executables are available at .

## Backgound

The computational discovery of DNA binding sites for previously uncharacterized transcription factors in groups of co-regulated genes is a well-studied problem with a great deal of practical relevance to the biologist, since such binding sites provide targets for mutational analyses (for reviews see [1-3]).

The position-specific variability of transcription factor binding sites makes their *de novo *identification challenging. Many computational motif finding methods are based on the observation that transcription factor binding sites occur more often than expected by chance in the upstream regions of the set of genes regulated by the same transcription factor [1]. The problem thus simplifies to the identification of overrepresented motifs in a given set of upstream sequences.

Motif finding programs rely on a search algorithm to optimize a motif model (an abstract representation of a set of transcription factor binding sites). Most recent programs represent motifs as position weight matrices (PWMs), which record the frequency of each base at every position in the motif. Other motif finding programs have relied on the use of consensus motif models (in which every base is represented by a letter of the 15-letter IUPAC code, which accounts for degeneracies as well as single bases) or *k*-mismatch motif models (in which a non-degenerate word with at most *k *allowed mismatches is used to represent the word). Regardless of the motif model used, a search for all overrepresented motifs of any length and degree of degeneracy leads to a dauntingly large search space. Thus, motif finding algorithms restrict their search space by using simplified motif representations, employing heuristic search strategies that are prone to local optima, or invoking additional parameters to limit the search space and thereby pass some of the optimization process off to the user [3].

Program parameters (such as motif length, number of occurrences and orientation) that cannot be reasonably specified by the user without prior knowledge about the true binding sites are referred to as nuisance parameters [4]. Selection of the correct settings for these parameters is a crucial step in motif finding, and is often assumed to be the domain of experts. In a recent evaluation, Hu and colleagues [4] compared the performance of five motif finders on a single prokaryotic genome, systematically exploring the effects of nuisance parameters, including expected motif length and number of occurrences. Every motif finder they tested was found to be sensitive to values used for these parameters. Guidance on the specific parameter settings to use for given motif finding situations is not provided in most publications presenting motif finders. Even assuming that optimal parameter settings exist for a motif finding program for each specific situation, for the typical biologist looking to identify motifs in a set of uncharacterized sequences, acquiring such expertise is an onerous task.

Nuisance parameters complicate the interpretation of performance comparisons as well. A recent large-scale performance comparison between thirteen different motif finding tools used expert knowledge in setting the parameters for every program [5]. Several of the programs contributing to the performance comparison were run with different parameter settings for each regulon, and in some cases, motifs were hand filtered as a post-processing step. Such performance comparisons evaluate not just algorithms but also the expertise of the users, making it difficult for a first-time user to select a motif finder on a principled basis.

A key result of the Tompa, *et al*. study was the finding that all of the motif finders had roughly the same average performance under a wide range of conditions and test statistics [5]. This finding was particularly notable because the motif finders studied employed a wide range of motif representations, scoring functions and search strategies and all were operated under the most favorable conditions possible. Although the average performance of the programs did not differ significantly, the authors found that, for each pair of programs, each program performed better than the other on some subset of the data [5]. Previous studies over smaller numbers of motif finders have found that no program clearly stands out as superior to the others and each program outperforms all others on some subset of the regulons [6-8]. This diversity of performance has led a number of authors to speculate that ensemble methods, comprising multiple motif finders, may lead to improvements in accuracy [1,5,8].

Ensemble methods, well known in the machine learning community [9], are typically composed of multiple methods comprising different search strategies (or the same search strategies with different initiation settings or random restarts) with a unified objective function. The final predictions are chosen from the ensemble of methods by a learning rule, which may be as simple as finding the maximum score from all the methods, or as complex as optimizing a weighted scoring scheme from among the methods. The construction of this learning rule is key to the performance of an ensemble learning method, as the performance of an ensemble method with an ineffective learning rule will be the average of the performance of its component algorithms. In this context, we note that Tompa *et al*. [5] found that, although every motif finding program tested had some regulons on which its performance was clearly superior, it was not possible *a priori *to predict which motif finder represented the best choice under any given set of conditions [5]. This observation serves to illustrate the challenges to the construction of an effective learning rule.

To the best of our knowledge, only one study to date has explored ensemble learning in motif finding. Hu, Li and Kihara [4] described a simple ensemble method wherein the component programs were random restarts of the same stochastic algorithm (such as Gibbs sampling or Expectation Maximization) and the learning rule was a voting scheme in which the results of each random restart cast a "vote" for which positions in the DNA sequence should be part of the final reported motif (hereafter, we refer to this as the HLK method). Under this scheme, the authors found that ensemble learning resulted in an increase in performance ranging from 6 to 45%. The HLK voting method provides a framework wherein a number of different motifs finders can be combined under the heuristic that if several motif finders make the same (or overlapping) prediction, then that prediction is accurate.

Here we present a novel ensemble motif finder based on a different conceptual approach. Rather than randomly restarting the same search algorithm or comparing multiple search strategies that all search for the same global optimum (and are potentially vulnerable to the same local optima), our algorithm assumes that the "biological significance surface" primarily consists of three local optima, and that one of these peaks represents the global optimum. Thus, our ensemble uses three specialized algorithms whose search spaces restrict them to each of these three local optima (BEAM for non-degenerate motifs, PRISM for degenerate motifs and SPACER for bipartite motifs). We have previously demonstrated that the greedy search strategies employed by each of these methods allow them to reliably search their respective motif domains without the use of nuisance parameters, as the algorithms themselves efficiently optimize the parameters that are typically forced on the users [10-12].

The results of these component algorithms are then combined using a learning rule that is simply the maximum score returned by each component algorithm. To make comparisons possible, the motif scores returned by each algorithm are penalized according to the complexity of the motif. The resulting ensemble algorithm, SCOPE, has no nuisance parameters and performs significantly better than its component algorithms. In addition, we find that SCOPE performs favorably compared to a diverse range of existing methods and is robust to the presence of extraneous sequences in its input.

## Results

### Algorithm

SCOPE takes as input a set of sequences ***U ***that are upstream of a set of genes ***G ***that are thought to be coregulated. The ultimate goal of a motif finder is to identify the specific subsequences ***Û ***in ***U ***that act as binding sites for the transcription factor(s) that regulate ***G***. In practice, sets of binding sites are represented using a *motif*. We have found that simple consensus motifs over the full IUPAC alphabet (a 15-letter code consisting of the bases A,T,C,G and all possible combinations) provide enough representational power to adequately describe ***Û***, while still allowing for an efficient search [3,4]. While alternative representations, such as position weight matrices (PWMs) are more expressive, their heuristic searches are prone to local optima and often do not perform well in practice [3,4,11-13].

**Figure 1:**
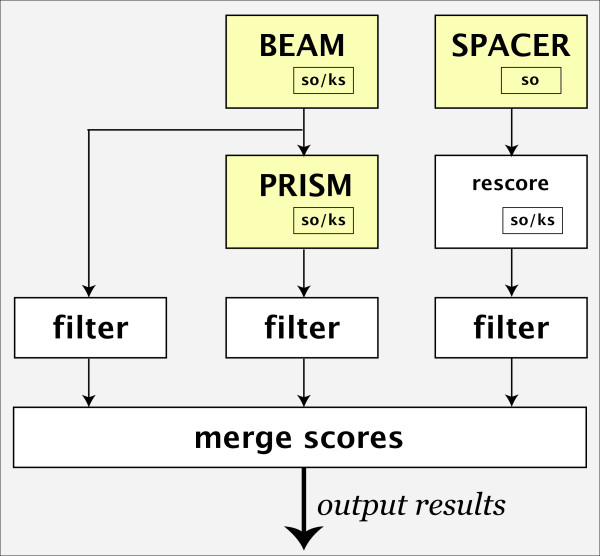
Flow diagram for SCOPE. BEAM and SPACER are run independently; PRISM runs on the top 100 motifs output by BEAM. For yeast (whose upstream regions are standardized to 800 bp), BEAM and PRISM use the overrepresentation-KS objective function (so/ks), while SPACER's slower running time requires the simpler overrepresentation objective function (so). The top 5 motifs from SPACER are rescored using the combined objective function. For bacteria and *Drosophila*, upstream regions are defined to be the intergenic region upstream of each gene; thus, the KS objective function is not used. The results of each program are sorted by *Sig *and lower scoring motifs that substantially overlap higher scoring motifs are removed. The filtered lists of motifs from the three programs are finally merged by *Sig *score. Repetitive motifs are identified and removed during all stages.

SCOPE has three component algorithms, BEAM, PRISM and SPACER, which search for non-degenerate, short degenerate, and long, highly degenerate and "gapped" motifs, respectively (Figure 1). Each motif is scored considering one or both strands and the motif is marked to indicate which calculation scores higher. The results of the three algorithms are merged and sorted. Artifactual motifs, whose significance can be accounted for by higher scoring motifs that they overlap, are identified and removed (for details, see Additional file 1, section S1).

Each of SCOPE's three component algorithms seeks to maximize the same objective function over a different class of motifs. Let *M *be a random variable over the full space of IUPAC words. The statistical significance *p*(*M = m*) of a particular word *m *is determined by the distribution of *M *over the entire space of upstream sequences in the given species. In general, we seek to maximize -log(*p*(*M = m*)). All values of *M *are not, however, equally likely *a priori*. For example, it is quite likely that there exists an extremely long sequence that is entirely unique to ***U***. Such a unique sequence would appear to be highly significant, until we consider that we have in effect searched all possible sequences until we found one that is unique. To correct for this *multiple hypothesis *testing problem, van Helden *et al*. [14] proposed using a Bonferroni correction, in which *p*(*M = m*) is penalized by the number of motifs *N *of length |*m*|:

*Sig *= -log(*p*(*M *= *m*)·*N*).

Thus, if *m *= "ACGT", *N *= 4^4^. We employed this same definition of *Sig *for BEAM, our algorithm that searches for non-degenerate motifs [10]. Defining *N *for degenerate or bipartite motifs raises a significant conceptual challenge. Van Helden *et al*. [14] chose to use the same definition, but limited their search to a small number of degenerate bases. In contrast, we have proposed that all characters should not be treated equally, but should be penalized in proportion to the information provided by them [11,12]. By this logic, "ACGT" will not be penalized differently from "ACNNNNGT", as both have the same number of bases that contribute any information to protein-DNA binding. Building on this intuition, one can argue that the characters "A" and "not-A" (IUPAC character "B") are roughly equivalent, while "A or G" (IUPAC character "R") is different from "A" as there are six ways to define a combination of two bases, while only four ways to define a combination of one base or three bases. For motif *m *= *m*_1_*m*_2_...*m*_*n*_, we can therefore define

N = ∏ Choose(4, |*m*_*i*_|),

where |*m*_*i*_| is the number of DNA bases covered by the IUPAC character *m*_*i*_. In the case were both orientations of the motif are considered, this number is adjusted to account for palindromes. The resulting *Sig *score thus penalizes motifs based on their length and degeneracy, enabling fair comparisons to be made between different motif classes.

### Testing

#### Evaluation of objective functions used by SCOPE

Each component algorithm in SCOPE efficiently searches its restricted search space, keeping SCOPE's runtime low (average runtime on our datasets were about one minute). This efficiency allowed us to explore several objective functions for scoring the statistical significance *p*(*M *= *m*) of motifs. These objective functions were as follows: position bias (based on the Kolmogorov-Smirnov, or KS, statistic), overrepresentation (a Poisson-based measure based on how often a motif occurs in ***U***) and coverage (a Poisson-based measure based on how many upstream sequences contain the motif). For precise definitions, see Methods.

**Figure 2:**
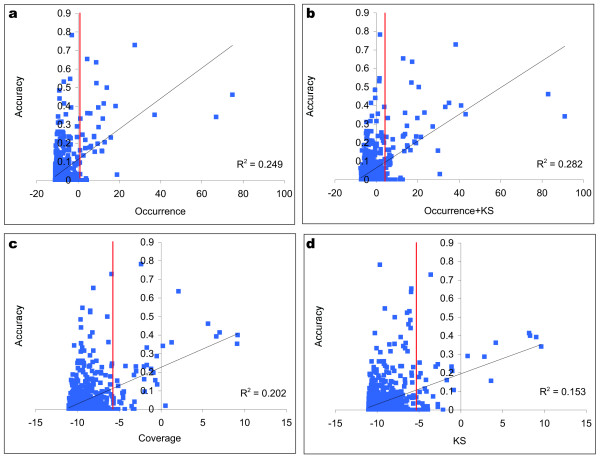
Correlation between accuracy and *Sig *scores. Non-degenerate 6-mers from *S. cerevisiae *were scored according to *Sig *scores based on (**a) **Overrepresentation, **(b) **Overrepresentation-KS, **(c) **Coverage and **(d) **KS metrics of statistical significance. The 6-mers were randomly sampled from both the upstream regions and the known binding sites to ensure coverage or a wide range of accuracy. The x-axis plots the Bonferroni-corrected and log_2 _transformed *Sig *score for each metric. The red lines indicate the 95th Sig percentile.

To establish which objective function (or combination of functions) was most suitable, we tested each objective function independently of SCOPE, using a subset of the *S. cerevisiae *dataset. The measure used to assess the biological relevance of a motif was *accuracy*, a measure of the nucleotide level overlap between a motif and the known binding sites (for details see Methods). From each regulon from the SCPD database [15] we selected ten six-mers at random from the upstream sequences and ten six-mers at random from the collection of known binding sites for that regulon. For each of these sampled six-mers, we calculated accuracy with respect to the known binding sites. We also calculated the *Sig *score for each six-mer, using four objective functions (KS, overrepresentation, coverage and combined KS-overrepresentation). We then plotted *Sig *versus accuracy for each objective function, to determine which objective functions correlated most strongly with biological relevance (Figure 2).

These plots demonstrate that overrepresentation is a closer approximation to biological relevance than coverage or KS alone. Adding KS to overrepresentation modestly improved the correlation by 13% (as compared to overrepresentation alone) to R^2 ^= 0.28. To assess the degree of class separation achieved by the two objective functions, we ranked the sampled six-mers by *Sig *score, and calculated the percentage of motifs with high *Sig *scores (in the 95^th ^percentile and above) that possessed a reasonable degree of overlap with the known binding sites (accuracy ≥ 0.10). By the overrepresentation measure, 74.4% of high scoring motifs had accuracy = 0.10, while 79.1% of high scoring motifs by KS-overrepresentation had accuracy ≥ 0.10.

This analysis suggests that more complex objective functions may provide a better estimate of biological significance than the overrepresentation objective functions commonly used. We thus chose to run SCOPE using the overrepresentation-KS combined objective function on the *S. cerevisiae *dataset, in which the upstream regions are of fixed length. We used the overrepresentation objective function for the other species, as our upstream definitions for those species were of variable length due to the available annotations. Because identifying the genomic positions of highly degenerate bipartite motifs is prohibitively slow, initial rankings of motifs for SPACER were computed using the overrepresentation objective function, and the overrepresentation-KS objective function was used only to produce the final ordering and scores. Although the KS objective function is computationally expensive (linear in the frequency of the motif in the genome), the SCOPE algorithms all aggressively limit the search space, thereby making the use of this objective function – and exploration of other complex objective functions – possible.

The surprisingly low correlations between *Sig *and accuracymay indicate that the objective functions employed by motif finding programs are only a first approximation to biological significance. Indeed, previous studies have reported little or no correlation between the significance measures of various motif finders and measures of accuracy [4,16]. Further research into more biologically accurate objective functions may yield better performance for motif discovery algorithms.

#### Evaluation of SCOPE performance and ensemble learning scheme

**Figure 3:**
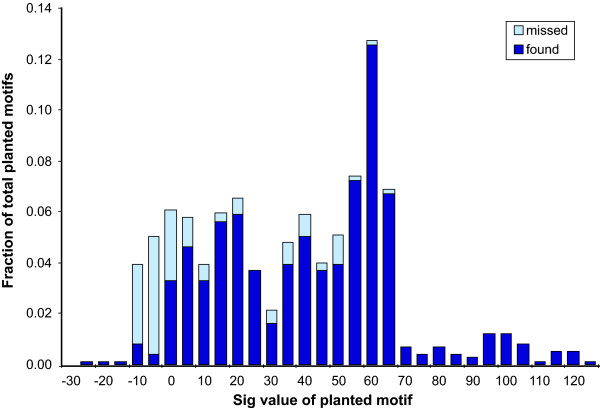
Performance at different overrepresentation *Sig *values on synthetic data. A motif was "found" if the top scoring motif returned by SCOPE overlapped the planted motif by at least 50%. Different *Sig *values were achieved by varying the number of upstream regions, the number of motifs per upstream region, and the number of extraneous upstream regions without planted motifs. A *Sig *value of 0 implies that one motif of that significance is expected by chance.

We first assessed the performance of the optimized SCOPE framework on synthetic datasets (for details, see Additional file 1, section S2). SCOPE performed well on the synthetic datasets, correctly identifying 92% of planted motifs that are over-represented relative to background (those motifs with a *Sig *score of greater than 5; Figure 3).

While synthetic test sets are useful in algorithmic development and initial testing, the results of such tests must be taken with a grain of salt, as they are highly dependent on the model used to generate the test sets [6]. We therefore tested SCOPE on an extensive array of regulons with known binding sites (for details of datasets, see Additional file 1, section S3). We ran SCOPE on each regulon and, following the scoring methodology used by Sinha and Tompa [6], we computed the accuracy for each of the top three motifs reported by SCOPE against the known binding sites. The motifs reported by SCOPE overlap to a large extent with the published *cis*-regulatory elements (as discussed in Additional file 1, section S3, a difference of one base pair length between the reported motif and the published *cis*-regulatory element results in an expected accuracy of about 0.25). SCOPE was run on 78 regulons from *S. cerevisiae, B. subtilis, E. coli *and *D. melanogaster*. On these datasets, SCOPE's average accuracy was 0.28, 0.29, 0.16, and 0.08 respectively.

**Figure 4:**
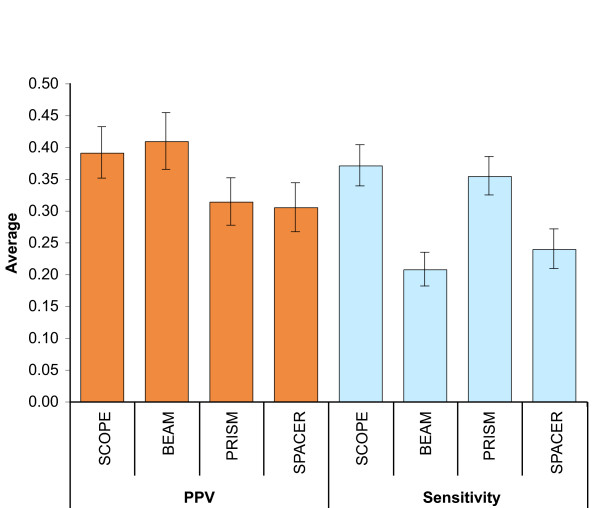
Average and standard error of sensitivity and PPV for the component algorithms of SCOPE on all 78 regulons. Bars represent standard error.

SCOPE's reported accuracy was significantly higher than any of its component algorithms (Table 1). Indeed, we found that SCOPE increased accuracy by 31–44% over BEAM, PRISM or SPACER alone. This improvement was achieved by combining BEAM's high *positive predictive value *(PPV) with PRISM's high *sensitivity *(Figure 4). Sensitivity is defined here as the fraction of the known binding sites (at the nucleotide level) predicted by the motif finder, and PPV is defined as the fraction of nucleotides predicted by the motif finder that correspond to the known binding sites (see Methods for details).

**Table 1:** Summary results for performance comparisons between SCOPE and its component algorithms, on all regulons. A "Win" is a regulon for which a program had the highest accuracy and that accuracy was at least 0.10. Programs in a two-way tie are credited with 0.5 wins each, so by construction, SCOPE can at best share a win with one of the other programs. A perfect winner-take-all ensemble method would have the same number of wins as all the component algorithms combined. A "clear win (loss)" is a regulon for which SCOPE's accuracy was at least 0.10 higher (lower) than the other program. The p-value reported for the paired t-test was Bonferroni-corrected to account for multiple (three) comparisons.

	**SCOPE**	**BEAM**	**PRISM**	**SPACER**
Average	0.24	0.17	0.18	0.17
Stderr	0.02	0.02	0.02	0.02
Wins	20	13	11	17
scores ≥ 0.50	8	8	6	5
scores ≥ 0.33	21	15	14	14
scores ≥ 0.20	39	23	23	26
Regulons returned	78	78	78	78
clear win for SCOPE vs	-	28	18	19
clear loss for SCOPE vs	-	6	2	3
t-test p-value	-	0.002	0.002	0.004

An ensemble motif finder with a learning rule that is no better than random will provide an accuracy that is equal to the average of its three component algorithms. To provide a basis for evaluating the performance of SCOPE's learning rule, we constructed an ensemble learning method (referred to here as BASELINE) from the results of BEAM, PRISM and SPACER, by randomly selecting one of the accuracies from these three programs for each regulon. Over 120,000 trials, BASELINE's average performance on this dataset was 0.176 with a standard deviation of 0.013. BASELINE's average score never exceeded that of SCOPE (*p *< 8.25 × 10^-6^). When compared to its component algorithms, SCOPE picked the highest accuracy motif in 66% of the cases (as opposed to 33% for a random selection between three algorithms). These results suggest that SCOPE's learning rule is highly effective, though it may certainly be improved further.

Of course, SCOPE's learning rule is extremely simple, and more complex learning rules may allow SCOPE to approach its theoretical upper bound. One rule that may prove effective is to weight the output of each algorithm according to (for example) the frequency of occurrence of each class of motif (non-degenerate, short degenerate or long degenerate) in the species or by learning the appropriate weights on a representative training set, creating, in effect, a Naïve Bayesian Network. The training of a more complex learning rule must, however, be performed in a cross-validation framework, and the size of the available dataset of regulons will place a practical limit on the complexity of the learning rule that can be devised.

#### Comparison with other motif finding programs

To provide a frame of reference for SCOPE's performance, we ran ten other popular motif finders on these datasets (for details and references see Table 2). We ran all programs directly from their websites, leaving all parameters at their defaults. The only parameter that we specified (where available) was the species from which the background sequences were derived. Thus, the results of this performance comparison may be interpreted as a comparison against other motif finders when those motif finders are run using their default values.

**Table 2:** Motif discovery algorithms used in the performance comparison. Nuisance parameters are parameters that cannot be precisely defined without knowledge of the true binding sites (such as motif length, number of occurrences and orientation). For MotifSampler and wConsensus, the lower part of the range indicates required parameters, while the upper part indicates the total number of parameters, including "power user" parameters that the program authors stress should typically be left as default. Motif model abbreviations: cons = consensus; PWM = position weight matrix; mis = consensus with predefined number of allowed non-position-specific mismatches.

**Program**	**# Nuisance Parameters**	**Motif Model**	**Search Strategy**	**Citation**
Oligo analysis (RSAT)	3	cons	Exhaustive enumeration of short and bipartite oligos. Clusters overlapping motifs. Uses a binomial approximation to the hypergeometric score, similar to the overrepresentation objective function.	[14, 33, 34]
Yeast Motif Finder (YMF)	2	cons	Exhaustive enumeration of short and bipartite oligos. Alphabet is {ACGTYR}. Uses the Normal approximation to the hypergeometric function, similar to the overrepresentation objective function.	[35]
AlignAce (AA)	2	PWM	Gibbs sampling to optimize a Maximum a Posteriori (MAP) score.	[36]
MotifSampler (MS)	3–5	PWM	Gibbs sampling with higher order Markov model.	[37]
BioProspector (Biopros)	7	PWM	Gibbs sampling with higher order Markov model. Designed for long and bipartite motifs common in prokaryotes.	[16, 38]
MEME	4	PWM	Expectation Maximization over a modified information content.	[39]
Improbizer (Imp)	8	PWM	Expectation Maximization. Uses 2nd order Markov model and optionally accounts for positional restrictions using a Gaussian model.	[40]
MITRA	1	mis	Tree-based search for long bipartite motifs with many mismatches. Uses a hypergeometric score similar to the overrepresentation objective function.	[41]
wConsensus (wCons)	1–13	PWM	Greedy enumeration to maximize information content. Infers motif length.	[42]
Weeder	4	mis	Bounded enumeration using a suffix tree. Tries all motif lengths from 6–12.	[43]

SCOPE has no user-adjustable parameters, although its component algorithms do contain a number of internal parameters ("hyperparameters") that govern their search over common nuisance parameters. On synthetic datasets, we found SCOPE's component algorithms to be quite robust to the settings of these hyperparameters. We have therefore fixed those parameters to reasonable values and do not expose them to the user [10-12]. This construction means that SCOPE can only run in a default configuration.

**Figure 5:**
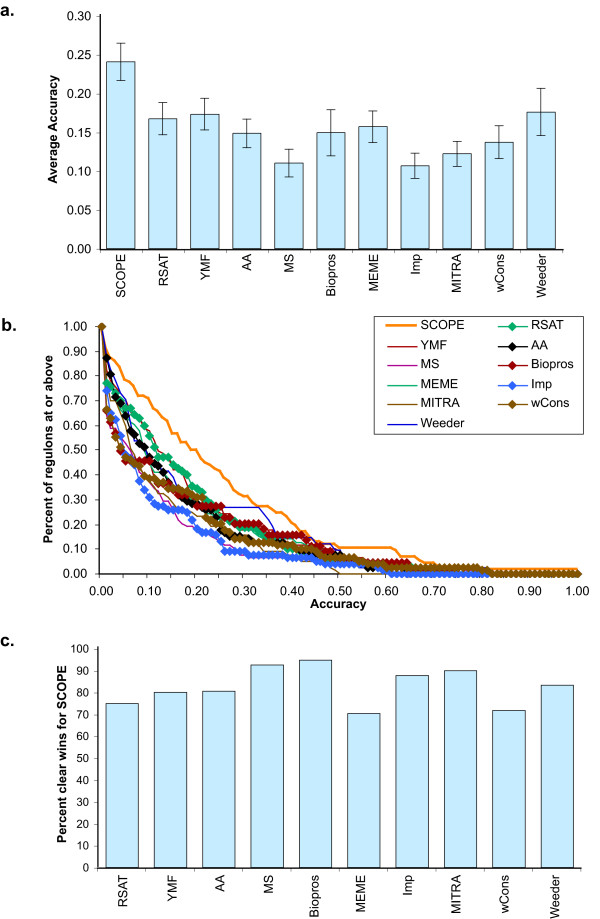
Performance comparisons. (**a**) Mean and standard error of accuracy for each of 78 regulons. (**b**) Cumulative distribution of accuracy for each program. (**c**) Fraction of regulons with a clear outcome (margin of difference in accuracy between two programs was greater than 0.10) won by SCOPE. Program abbreviations and details in Table 2; performance details in tables S1 and S2 in Additional file 1.

We compared the motif finding programs using the criteria set forth in Sinha and Tompa, including average accuracy and the number of total wins (highest accuracy on a regulon, where that accuracy is at least 0.1) [6]. On this dataset, SCOPE had the highest score by both criteria (Figure 5a). The cumulative distribution of accuracy shows that SCOPE had the most high-scoring motifs at every level (Figure 5b). When we looked at the number of clear head-to-head wins (such a win is taken to occur when the difference in accuracy between SCOPE and another motif finder is greater than 0.1 [6]), we found that SCOPE scored a clear majority (82%) of clear head-to-head wins (Figure 5c). The average accuracies of BEAM, PRISM and SPACER on this dataset were similar to those of the ten other programs.

A formal statistical analysis found that SCOPE's performance margin over the other motif finders run on this dataset was statistically significant at p < 10^-5 ^(for details, see Additional file 1, section S3). Corroborating the results of previously published performance comparisons [1,4-7], none of the other programs showed a statistically significant difference relative to the other nine. Similarly, none of SCOPE's component algorithms outperformed the other ten programs on this dataset by a statistically significant margin.

**Figure 6:**
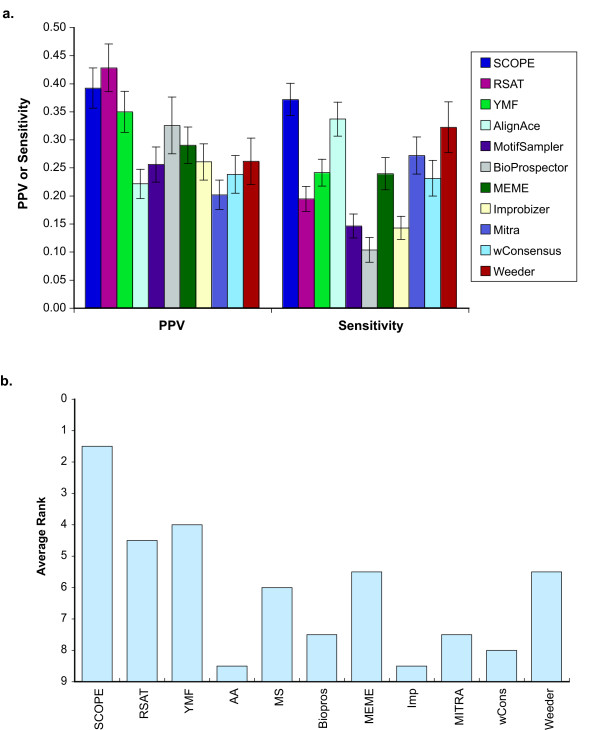
(**a**) Average and standard error of sensitivity and PPV for each program on all 78 regulons. In cases where the program failed to return a result, the sensitivity is 0 and the PPV is undefined. Cases where a program did not support the species were not included. (**b**) Ranks on this plot were computed by taking the average of sensitivity and PPV ranks for each program.

SCOPE's high accuracy was a reflection of both high PPV and high sensitivity (Figure 6a; see Methods for a precise definition). By these measures, SCOPE was the only program that scored highly in both sensitivity and PPV (ranking first and second respectively). In contrast, none of the other motif finders that performed well by one criterion performed well by the other, as shown by the average ranks for each motif finder over both sensitivity and PPV (Figure 6b).

#### Performance in the presence of extraneous upstream sequences

In practice, microarray co-expression data are often used to identify genes in a particular regulon. This approach identifies genes that are either directly or indirectly regulated by the transcription factor of interest. Therefore, sets of genes identified from co-expression data may often contain multiple extraneous upstream sequences. Adding sequences that do not contain binding sites decreases the signal-to-noise ratio, making motif finding more difficult [4].

**Figure 7:**
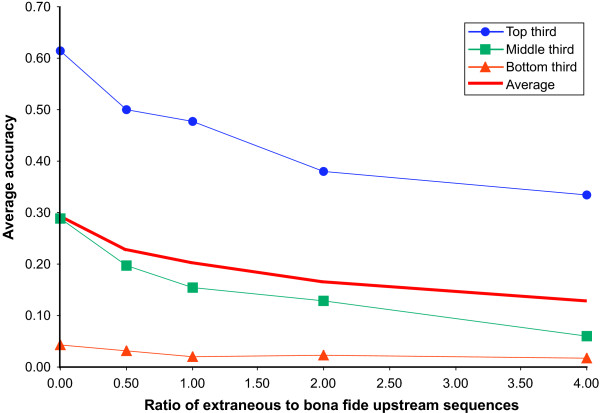
Robustness of SCOPE performance on *S. cerevisiae *regulons containing extraneous upstream sequences. Increasing quantities of randomly selected upstream regions were added to each regulon. The bold red line is the average across all regulons, while each of the other lines represent the performance of SCOPE on one-third of the total *S. cerevisiae *regulons. The y-axis shows the average accuracy for each group of regulons. The x-axis shows the ratio of extraneous upstream sequences to *bona fide *ones.

We thus tested SCOPE's performance on regulons containing additional extraneous upstream sequences. For all 33 regulons in the SCPD dataset, we added randomly selected upstream *S. cerevisiae *sequences such that the total number of extraneous sequences was between 0.5 and 4 times the number of true upstream sequences in the regulon. SCOPE's accuracy on this dataset was remarkably stable in the presence of extraneous sequences. Figure 7 shows the aggregate results of this test, with the SCPD regulons divided into three groups based on SCOPE's accuracy on the true regulon. For each set of regulons, SCOPE's performance decayed gradually as increasing numbers of extraneous genes were added to the regulon. These results were consistent with the relationship between the *Sig *score and performance on synthetic datasets (Figure 2).

## Discussion

The field of motif finding is saturated with a large number of algorithms representing myriad search strategies, objective functions and motif models. Yet remarkably, performance comparisons consistently reveal disappointing performance for motif finders and fail to find any statistical significance between them. A brief survey of the per-regulon results of these performance comparisons yields two key observations: (1) there are many regulons for which a large number of programs find a small portion of the binding sites (though not necessarily the same portion); and (2) every program has a respectable number of "wins" (i.e. every program is the best existing program on some handful of regulons [1,4-8].

Such observations are common in many machine learning applications, and are the direct result of complex search spaces that force restrictions on either the search strategy or the representation of the solution space (in this case, the motif model used to represent the motifs). For example, YMF and RSAT are guaranteed to find the optimal solutions in their motif space (fixed-length motifs with limited degeneracies), but that space is limited to the point that optimality provides no clear advantage over the other methods. Conversely, the PWM-based methods have an apparently more powerful motif model [17], but their search strategies cannot guarantee optimality and often terminate at local optima.

The HLK ensemble method [4] successfully exploits the first key observation above. By running the same (stochastic) algorithm multiple times and using a voting method, those subsequences of the binding sites that are repeatedly reported become clear while the spurious bases are eliminated. Hu and colleagues report that this method increased accuracy and proposed that their approach may prove effective when running different algorithms as well [4]. The limitation arises, however, in regulons where only one program has a high accuracy and the others fail to find any portion of the binding sites. In such cases, it is likely that a voting-based ensemble will follow the crowd and fail to find the true binding site.

The second observation, that all motif finders win some number of regulons and often perform roughly the same on average, is broadly consistent with a theorem in the Machine Learning field referred to as the No Free Lunch Theorem [18,19]. Briefly, this theorem states that, averaged over all datasets, the performance of all search algorithms are exactly the same, with the corollary that two algorithms will have the exact same number of wins in relation to each other. In practice, this theorem argues for the use of specialized domain knowledge [20], where available, and may suggest that similar average performance across a diversity of approaches is an indication of the diversity of the datasets themselves. Thus, motif finders designed for one class of motifs will win on regulons containing those motifs, but will perform poorly on other regulons, while more general motif finders will tend to have more consistently mediocre performance.

In this light, SCOPE can be seen as leveraging the second key observation by embracing the No Free Lunch Theorem: rather than boost average performance by taking the average results of three general purpose algorithms, SCOPE uses highly specialized algorithms and assumes each will perform strongly on some regulons and weakly on others (and that the unified scoring metric can tell the difference). The working hypothesis is, in effect, that the local maxima are *predictable *(corresponding to one of three motif classes) and *exploitable *(we can find the local maxima in each class and choose whichever is higher). Consistent with this hypothesis, there was very little overlap among the component algorithms of SCOPE (each wins about 20 of the 78 regulons, with very few ties) and, by taking the maximum score from those three local maxima, SCOPE tended to choose the motif with the highest accuracy in a clear majority of the cases (66%, compared to 33% for a random learning rule). Furthermore, SCOPE not only significantly outperformed its components on this dataset, it also outperformed a number of general purpose algorithms that seek to find the global maximum in a single pass.

Of course, based on the No Free Lunch Theorem, SCOPE's performance averaged over all theoretically possible datasets will still converge to that of the other motif finding approaches (including random guessing). As the physical properties of transcription factors will inevitably constrain the structure of their binding sites, biologically relevant datasets comprise a subset of the space of all theoretically possible sequences. Our test set of 78 regulons was selected in a blinded manner (for details, see Additional file 1, section S3), thus these results suggest the generalizability of SCOPE's use of domain knowledge on biologically relevant datasets from these species.

These observations are not offered as definitive proof that there are only three classes of motifs; rather, they show that power can be gained by identifying distinct motif classes and combining specialized algorithms with a unified scoring rule. It is possible that more power could be gained by identifying other distinct motif classes and adding algorithms that explicitly search for those classes. For example, Zinc finger transcription factors have been demonstrated to bind three triplets of nucleotides which overlap at their third base positions [21]. This observation could be leveraged by a search algorithm that explicitly searches for motifs matching this unique structure. Thus, all nondegenerate triplets in a set of upstream regions could be scored and the highest-scoring triplets combined into a single five-mer with a two-base degeneracy (corresponding to the IUPAC characters R,Y, W, S, K or M) at the middle position. The highest-scoring five-mers could then be combined with the highest scoring triplets to generate a seven-mer with two-base degeneracies at positions three and five. Provided the appropriate Bonferroni correction is applied for this new class of motifs, these motifs may be easily compared with the results from BEAM, PRISM and SPACER, thereby extending the SCOPE ensemble to include a fourth class of motifs. We note, however, that as more methods are added to SCOPE, it will be increasingly difficult to devise a scoring metric that can accurately choose the best result from among the components.

SCOPE may also serve as a complementary approach to the HLK method. For example, the parameters of many methods can be set to search for specific classes of motifs (such as bipartite versus non-bipartite motifs). Thus, analogous to the ensemble method described in this paper, one may build a hierarchical ensemble that first searches each motif class by the HLK method using a number of algorithms or random restarts, and then uses the SCOPE method to choose the best result from among the motif classes. One constraint associated with such an approach is the run-time. A second constraint associated with a hierarchical ensemble learning method is the multiplicative increase in the number of parameters associated with it, though this problem may be ameliorated by the use of parameter-free algorithms that employ restricted search spaces.

An important factor to consider when taking the best of multiple runs is the relative size of the search space. Certainly to maintain statistical validity, some correction must be made for multiple hypothesis testing. Furthermore, the effects of multiple testing may bias the results in favor of one of the component algorithms. To ensure statistical validity and avoid such a bias, we developed a simple Bonferroni-like correction, which penalized every proposed motif proportional to its length and degree of degeneracy, resulting in a modest improvement of 8% in SCOPE's accuracy.

Although our test set of 78 regulons gave us enough power to find significance between SCOPE and its components or other algorithms, it did not provide enough power to disentangle the effects of small improvements (such as the Bonferroni correction, the objective function that takes position bias into account, or scoring motifs based off one or both strands), especially in the rigorous cross-validation framework necessary to decipher precisely which aspects contribute significantly to the performance. Nevertheless, as larger datasets become available, SCOPE's efficient search strategy makes it an ideal platform for exploring the effect of focused improvements to the motif finding approach described, such as the use of complex objective functions that may better approximate biological significance.

The comparisons to other motif finding programs in this study are provided to place SCOPE's performance in the broader context of the motif finding field, particularly when viewed from the standpoint of the practicing "bench" biologist. Any performance comparison must be interpreted with caution, since the results are highly dependent on the dataset used, the conditions of the testing and the metrics used for evaluation. With this in mind, we sought to evaluate a wide representation of motif finders on a large number of regulons using performance metrics consistent with previous studies [6,7]. To the best of our knowledge, this dataset represents the largest set of biologically relevant regulons used for performance comparisons to date. Whereas previous performance comparisons attempt to optimize the parameters of the programs in question [4,6,7] or allow expert users to tune their own programs and manually filter both the input and output [5] we intentionally made our comparisons between programs without manually optimizing any parameters for any species so as to emulate typical use conditions. Our comparison thus complements the recent large scale study of Tompa *et al.*, who gauge performance under optimal conditions on semi-synthetic data sets [5], as well as the study of Hu *et al.*, who explore the effect of parameter optimization on a handful of popular motif finders [4]. Although the present philosophy of performance comparison implicitly benefits SCOPE, which has no parameters to optimize, it is arguably the most relevant comparison possible for the typical biologist. Although previous studies have shown the potential importance of choosing parameters carefully [4,6], we note that the results we obtained under default settings were quite similar to those reported in previous studies (for details, see Additional file 1, section S3). Arguably, systematic parameter optimization for each of these programs may well yield higher accuracy scores than those reported here. However, in order to avoid the pitfall of overfitting to the dataset, such parameter optimization must be performed using cross-validation or some other resampling technique [9,22,23].

We note that all the motif finders tested, including SCOPE, performed poorly on the *Drosophila *dataset. Although SCOPE had the highest accuracy on this dataset, that accuracy was significantly less than on the bacterial and yeast data. Especially poor performance on *Drosophila *was also reported in the Tompa *et al*. performance comparison, indicating that this difficulty is not limited to the current dataset [5]. One possible cause of poor performance in this study is that the "regulons" are derived from enhancer regions defined in an earlier computational paper [24]. Whereas a background set of promoter regions is easy to identify, it is not clear how to define a reasonable genomic sample of enhancers. Thus, the background sequences used by SCOPE and the other programs may not be representative of the "true" background model of enhancers, leading to inaccurate statistics. The persistently poor performance of motif finders on *Drosophila *regulons thus highlights the importance of using well-defined background sequences to calibrate the statistics of the objective functions being optimized. Recently, algorithms have been reported that predict enhancer regions on a genome wide scale [[24-26][27,28]]. It is possible that using such algorithms to define a collection of background enhancer sequences may improve the performance of SCOPE, as well as that of the other motif finders, on *Drosophila*.

## Conclusion

Ensemble methods hold the potential for providing improvements in motif finding accuracy without resorting to the use of additional data (such as phylogenetic information or characterization of the domain structure of the transcription factor), which are not always available. Typically, ensemble learning methods are plagued with certain liabilities, such as increased runtimes, logistical complexity and a multiplicity of nuisance parameters, all of which grow with the number of programs run. In the machine learning field, ensemble methods have coexisted for many years with non-ensemble methods, with no clear superiority having been established between the two.

SCOPE serves as a proof-of-concept, demonstrating an efficient and effective approach to ensemble-based motif finding. By dividing the search space into tractable domains, SCOPE mitigates the potential liabilities associated with ensemble methods, resulting in a program that is capable of finding *cis*-regulatory elements of arbitrary length, degree of degeneracy, motif orientation and frequency of occurrence. Its strong performance, rapid runtime and freedom from nuisance parameters make it a simple and effective tool for the biologist.

## Methods

### Accuracy, Sensitivity and Positive Predictive Value

Each algorithm's accuracy for each regulon was measured via the *Phi *score (also referred to as nucleotide level performance coefficient, or nPC, in previous performance comparisons [4-6,11]. This metric, first proposed by Pevzner and Sze [29], measures the degree of overlap between the actual instances of two motifs (or sets of motifs) *m*_1 _and *m*_2 _in the set of co-regulated upstream sequences. The *Phi *score can be defined as follows: let *U *be a unique numbering of all the bases in the upstream sequences of a given gene set, and *I*_*U*_(*m*) ⊆ *U *be the set of bases that are covered by actual instances of *m *in *U*. *Phi *is then defined as the ratio of the number of bases occupied by the actual instances of both the motifs, to the total number of bases occupied by the actual instances of either of the two motifs:

Φ_*U*_(*m*_1_, *m*_2_) = [*I*_*U*_(*m*_1_) ∩ *I*_*U*_(*m*_2_)]/[*I*_*U*_(*m*_1_) ∪ *I*_*U*_(*m*_2_)].

This metric therefore takes both false positives and false negatives into account at the level of the individual bases that are actually covered by the motif. As in Hu et al. [4], we define *accuracy *to be the *Phi *score between the known and predicted binding sites. Changing the denominator of the *Phi *equation to be *I*_*U*_(*m*_*i*_) yields the sensitivity (if *m*_*i *_represents the true binding sites) or the positive predictive value (PPV, if *m*_*i *_represents the reported binding sites). See Additional file 1, section S3, for a discussion on the use of *Phi *score for measuring accuracy.

### Objective functions for Statistical Significance

In line with other motif finders, we have used statistical significance as a surrogate for biological significance. Since the latter cannot be defined without data that obviates the need for computational motif finding, objective functions that approximate biological significance are critical. In this section, we detail the objective functions we used and their effect on SCOPE's performance. For any motif *m*, each objective function provides a definition for *p*(*m*), the probability of observing a motif with the same sufficient statistics as *m *assuming a particular null model. This *p*-value is used in the computation of the *Sig *score (see Results).

#### Overrepresentation

The most common statistical test in motif finding is based on overrepresentation, which can be roughly defined as the probability that a motif *m *that is observed *C*(*m*) times in the regulon would occur at least *C*(*m*) times in a random collection of the same number of genes. In the context of consensus motifs, overrepresentation is expressed in terms of a multinomial model, in which each position *i *in each gene *j *is a random variable *X*_*ij *_that can take on any motif allowed by the particular motif model. The probability of seeing *m *at least *C*(*m*) times in the regulon can be approximated by the Poisson distribution:

*p*(*m*) = ∑_k≥C(*m*) _[(λ^*k*^*e*^-λ^)/*k*! ]

where λ is the expectation of *C*(*m*) with respect to the null motif distribution and the number of positions in the regulon. A detailed justification of this approach was given by Carlson *et al*. [11]. The expectation λ is most accurately modeled using Maximum Likelihood Estimators (MLEs) computed as the actual proportion of any given motif in the complete set of all upstream sequences in the genome [10]. These MLEs are implemented as lookups of exact substrings, which can be performed efficiently using a suffix array data structure [10-12].

#### Coverage

A simple modification to the overrepresentation objective function is *coverage*, which is identical to overrepresentation with the modification that *C*(*m*) is the number of upstream regions in the regulon that have one or more instances of *m *and λ, the expectation of C(*m*), is determined from the proportion of upstream regions in the genome that contain the motif. While this objective function prevents a single upstream region from dominating a motif's score, it fails to account for multiple instances of a binding site in a single gene that may arise due to cooperative binding.

#### Positional bias

Transcription factors often require their binding sites to be located in a restricted range relative to the start of transcription. One well known example is TBP (TATA-binding protein), which localizes the RNA polymerase complex by binding the TATA-box motif roughly 25 bases upstream of the transcription start site [30]. While other examples of binding sites with positional restrictions are well known, few motif finders incorporate position in their scoring function. In the case where all upstream regions are the same length, the Kolmogorov-Smirnov (KS) statistic provides a natural test for positional bias. The Kolmogorov-Smirnov (KS) statistic is a non-parametric statistic that measures the probability that two samples are drawn from the same distribution. Let *X *be the sample that we wish to compare to some reference sample *Y*. The KS statistic is defined to be the maximum absolute difference between the unbiased cumulative distribution functions of *X *and *Y*. The KS statistic has a well-defined distribution from which a *p*-value can be easily computed. Kuiper's variation was used to increase sensitivity in the tails of the distribution [31].

In the context of motifs, we defined the test sample *X *for a motif *m *to be the set of starting positions (with respect to transcription start sites) of *m *in the regulon. The reference sample *Y *is defined as the set of starting positions of *m *in all upstream regions in the genome. Thus, *p*_*KS*_(*m*) is a measure of how *m *is localized differently in the regulon than in the genome as a whole. It is also possible to define *Y *as the uniform distribution; however, we found that many motifs had non-uniform distributions throughout all upstream regions of the genome, possibly as an artifact of the non-uniform AT/CG distributions in upstream regions [32].

#### Combining overrepresentation and positional bias

Since overrepresentation and KS are independent, the probabilities can simply be multiplied together to yield the probability of randomly sampling a motif with a given degree of overrepresentation and positional bias.

### Motif orientation

Many transcription factors will bind motifs on either DNA strand. Others, such as the general transcription factor TBP (TATA-Binding Protein), require a specific orientation and will only function if bound to motifs on a specific DNA strand [30]. In scoring a motif *m*, a choice must therefore be made as to whether or not the reverse complement *m*^*R *^of *m *will be considered to be the same motif as *m*. Most programs assume motif orientation does not matter and so define *m *= *m*^*R*^. Such an assumption may be overly generous – as the TBP example above makes clear, the transcriptional machinery of a cell is clearly able to differentiate between the two strands. We thus chose to attach a flag to each motif, indicating whether or not the motif should be orientation-neutral. BEAM and SPACER thus enumerate and evaluate all motifs with both values of this flag. SCOPE reports that orientation does matter (i.e. *m *≠ *m*^*R*^) for 17% of the regulons in our biological test set.

## Availability and requirements

A user-friendly web server, source code and executables are available at the project website.

• **Project name: **SCOPE

• **Project home page: **

• **Operating system(s): **Platform independent

• **Programming language: **Java

• **Other requirements: **Java 1.3.1 or higher

• **License: **Free for academic use

• **Any restrictions to use by non-academics: **License required

## Authors' contributions

AC proposed the original method, designed the experiments and helped design the web front end. JMC implemented SCOPE, contributed to the methodology, and helped design the experiments and the web front end. AC and JMC drafted the manuscript. RSK managed the performance comparison. RHG conceived the overall outline of the study, provided funding, contributed to the methodology and helped design the web front end. All authors contributed to, read and approved the final manuscript.

## Supplementary Material

Additional file 1 Details of the algorithms, data sets and statistical analyses. This file contains the details needed to replicate the experiments and the statistical analyses, as well as an overview of the component algorithms. Click here for file
