# Supplementary material for: A novel ensemble learning method for de novo computational identification of DNA binding sites
Source: BMC Bioinformatics. 2007 Jul 12;8:249. doi: 10.1186/1471-2105-8-249 (PMC1950314; doi:10.1186/1471-2105-8-249)
Supplement: Additional file 1 — Details of the algorithms, data sets and statistical analyses. This file contains the details needed to replicate the experiments and the statistical analyses, as well as an overview of the component algorithms. [file 1471-2105-8-249-S1.pdf]

Details of the algorithm, data sets and statistical analyses

S1. Component Algorithms of SCOPE

SCOPE works by searching for three classes of motifs: non-degenerate motifs of arbitrary length, short degenerate motifs, and long degenerate motifs (including those with "gapped" binding sites). Each algorithm searches for the motif within its class that has the maximum *Sig* score. The final results are sorted by the *Sig* score [1, 2] and redundant motifs are removed. In this section, we briefly describe the algorithms that comprise SCOPE, as well as the specific filtering technique used to remove artifacts from the combined output. The interested reader is referred to our previous papers for algorithmic details and full theoretical and experimental justifications for each algorithm [1-3].

a. BEAM: finding non-degenerate motifs

BEAM [2], aimed at the identification of statistically significant, non-degenerate consensus motifs of any length, is based on a greedy beam search algorithm [4]. Existing methods in the literature guarantee optimality for short motifs of prespecified length [5], or seek to merge overlapping motifs from among the most significant short motifs [6]. In contrast, BEAM uses the heuristic that highly significant motifs (under any distribution) must contain significant submotifs of shorter length to construct significant motifs of arbitrary length (error bounds for this heuristic are discussed in detail in ref. [2]) BEAM first enumerates all short sequences (N-mers) of a given length l (starting at l=5), then extends the 500 highest scoring N-mers by one base and repeats the procedure for the new length. As only 500 N-mers are scored for each length, the algorithm is both efficient and likely to find the most significant motifs.

b. PRISM: generalizing to degenerate motifs

PRISM is based on a greedy search algorithm that generalizes each output motif from BEAM into a degenerate version [1]. For each base, PRISM identifies the single degenerate modification that maximizes the statistical significance, iterating until no further improvements to the statistical significance are found. Experimental results show that PRISM is highly effective at finding motifs of arbitrary degree of degeneracy, though its performance breaks down for excessively long degenerate motifs, included "gapped" motifs. In performance comparisons, PRISM outperformed nine popular motif finders on degenerate *S. cerevisiae* motifs [1].

c. SPACER: finding gapped and long degenerate motifs

SPACER extends the conceptual framework of BEAM and PRISM to bipartite and long, highly degenerate motifs [3]. SPACER first enumerates all motifs of the form A-S_n-B, where A and B are non-degenerate 3-mers and S_n is a spacer region of 2-11 bases. It then iteratively extends the A and B regions of the most significant motifs in a manner similar to BEAM. Next, SPACER uses PRISM to generalize the A and B cores of the highest scoring motifs. Finally, SPACER specializes the spacer region by taking all the non-degenerate putative binding sites in the regulon that match a given bipartite motif and iteratively merging them in a greedy fashion. On a test set containing 39 experimentally defined regulons (based on Zn(II)Cys6 bipartite fungal motifs and long, highly degenerate bacterial motifs), SPACER significantly outperformed a range of popular motif finding programs.

d. Filtering overlapping motifs

Substrings of *bona fide* binding sites yield trivially high scores by any statistical metric that correlates well with biological relevance. These artifactual motifs are filtered from the output of each of SCOPE's three constituent algorithms based on the degree of actual overlap in the group sequences. This is measured using the *Phi* score (the same measure used to define the accuracy when one of the two motifs is the set of true binding sites), which measures the percent overlap between the bases of two motifs in a given regulon. When two motifs are found to overlap by at least a fixed threshold, the lower scoring motif is discarded. The *Phi* score cutoffs used were determined empirically from the synthetic datasets (Section S2). These values were 0.01, 0.01 and 0.5 for BEAM, PRISM, and SPACER, respectively. SPACER's relatively high threshold is due to the long, highly degenerate nature of the motifs it reports.

The *Phi* score approach is an approximation to a more rigorous interpretation of the artifactual substring problem. If m_1 is the highest scoring motif, then the remaining sites can be considered independent only if we recompute the statistical significance *conditioned* on the observed distribution of m_1. Thus, the significance of all reported motifs must be recalculated by conditioning on the existence of those motifs that are more significant. This approach was described by Blanchette and Sinha [7] and implemented as the program Best Explanators, which is run as a post-processing step by YMF [5].

Although Best Explanators provides a rigorous framework in which artifactual, overlapping motifs can be identified, the computational requirements grow prohibitively burdensome for complex statistical metrics. The *Phi* score filter approximates this approach by

the assumption that the conditional probability p(m_i | m_j) differs from p(m_j) if and only if motifs m_i and m_j overlap in the regulon. Furthermore, it assumes that if m_i and m_j overlap, then p(m_i | m_j) will not be significant. While there exist transcription factors whose binding sites may overlap to some degree [8], such examples are rare and do not seem to justify the computational cost associated with the complex statistical metrics we employ. Our implementation of Best Explanators provided no observable difference to the *Phi* score method on the synthetic test sets. While we consider the *Phi* score thresholds to be fixed, the command line executable exposes these parameters so that users may run Best Explanators if so desired.

e. Filtering repetitive motifs

Repetitive regions in upstream sequences (such as AT repeats) cause problems for most statistical metrics, as they are highly autocorrelated. Since autocorrelation violates the independence assumption inherent in most statistical metrics, repetitive motifs tend to have artificially high scores. A common solution is to mask repetitive regions from the input sequences. To minimize the burden on the user, we incorporate this masking into the individual algorithms by removing repetitive motifs after each iteration of BEAM and SPACER's non-degenerate motif search. Thus, we remove all motifs with a periodicity of 1 or 2 (e.g., poly-A and poly-AT motifs).

S2. Validation on Synthetic Data Sets

Synthetic data sets were constructed using sets of randomly selected 800 bp upstream sequences from *S. cerevisiae* as background. For each data set, one or more *cis*-regulatory element(s) were selected at random from the TRANSFAC database [9], and planted into a collection of randomly selected upstream sequences. We constructed 120 synthetic datasets, which varied widely in number of genes in each dataset, number of *cis*-regulatory elements planted and number of noisy genes (upstream regions without planted motifs) present. All development and parameter optimization was performed using these synthetic regulons.

We assessed SCOPE's performance using the synthetic datasets and the overrepresentation metric. We measured performance on these datasets using Positive Predictive Value (PPV) and False Negative rates (calculated via textual comparison, based on comparing the highest-scoring motif returned by SCOPE against the planted motif). In this evaluation, we considered 2 motifs to be *textually equivalent* if one is a subset of the other and is at least half the

length. That is, if m_1 is smaller than m_2, then m_1 and m_2 are equivalent if and only if m_1 is a substring of an instantiation of m_2, and the length of m_1 is at least half that of m_2.

We tested SCOPE's sensitivity to a wide range of *Sig* scores, by varying the number of times a motif was planted in each upstream region, as well as the number of upstream regions that did not contain any planted motifs (Figure 3 of the main paper). At *Sig* values greater than 15, corresponding to strongly over-represented *cis*-regulatory elements with low internal variation, SCOPE found 93% of all planted motifs. At *Sig* values between 0 and 15, corresponding to weakly over-represented *cis*-regulatory elements with low internal variation, SCOPE found 81% of all planted motifs. Finally, at *Sig* values at or below 0, SCOPE found 31% of all planted motifs. A *Sig* score of 0 indicates that we would expect one motif of that significance to exist in a random regulon.

S3. Validation on Biological Datasets

a. Test Regulons

Previous studies have found that motif finders perform best on synthetic datasets generated under a model similar to that on which the motif finder is based [10]. Rather than bias our experiments in favor of SCOPE (or any other motif finder), we chose a large range of known regulons from several species.

We tested SCOPE's performance against experimentally determined regulons from *S. cerevisiae*, *B. subtilis*, *E. coli*, and *D. melanogaster*. Each regulon consisted of a set of upstream regions of genes that had been previously demonstrated to be bound by a given transcription factor based on direct biological evidence. We refer to each regulon by the name of the regulating transcription factor. The databases used for each species were as follows:

S. cerevisiae: Saccharomyces cerevisiae Promoter Database ([8], http://rulai.cshl.edu/SCPD/).

B. subtilis, E. coli: PRODORIC (Prokaryotic Database of Gene Regulation [11], http://prodoric.tu-bs.de/).

D. melanogaster: Interactive collection of *cis*-regulatory modules from Drosophila ([12]; https://webfiles.berkeley.edu/~dap5/data_04/appendix2.htm).

All available regulons for each organism with five or more binding sites reported (as of March 2004) were compiled in our dataset.

b. Measuring accuracy using the *Phi* score

As described in Methods, we measure a motif's accuracy by computing the *Phi* score between the instances of the motif in the upstream regions and the instances of the known binding sites in those upstream regions. Using a scoring metric like the *Phi* score that directly compares the predicted and published binding sites without regard to motif representation is advantageous for a number of reasons.

The obvious advantage is that comparisons between predicted motifs and published motifs can be made in the same manner for all motif finding programs. Additionally, comparing the list of predicted binding sites against the list of published binding sites directly sidesteps the problem of spurious binding sites. (Spurious binding sites arise because motif models are inherently imperfect, meaning that a motif model fitted to include all known binding sites will also describe a number of motifs that do not correspond to actual binding sites. For example, if the known binding sites are ACGT and AGCT, the consensus representation will be ASST, which describes ACGT, ACCT, AGCT and AGGT, two of which are spurious.)

Other metrics for performance comparisons have been used previously, such as mutual information [13] and textual overlap [14], for PWM and consensus representations respectively. Both metrics are limited by their assumption of equal lengths for all binding sites. Transcription factor binding sites differ widely in length, sometimes even for the same transcription factor, and mutual information and textual overlap remain undefined in these cases.

Papers that use textual overlap as their main metric typically report high performance via this metric (see, for example [14]), in contrast to the low accuracy reported here and in other papers that use *Phi*-based accuracy as a performance metric. We view this disconnect as an indication that textual overlap can be a misleading metric of similarity, as two motifs that look very similar can describe a very different set of binding sites. For example, consider the motif pairs AT<u>C</u>AGTCG and AT<u>T</u>AGTCG. Although the motifs are almost identical upon visual inspection, their actual occurrences in the genome do not overlap at all. The textual overlap reported between this motif pair is 0.88, while the *Phi* score is 0. As a more general example, if consensus motif m_1 is a substring of m_2, it can be shown that the expected *Phi* score of m_1 and m_2 is close to 0.25^d, where d is the difference in length between the two motifs (assuming equal base frequencies). Thus, if m_1 is two bases shorter than m_2 but otherwise identical, visual inspection would suggest a reasonable match, while the expected *Phi* score would be approximately 0.06. Indeed, in a recent performance comparison, Hu *et al.* [14] found that low accuracy translated to

relatively high "motif level success rates," a measure of the percentage of regulons for which some portion of the real binding sites were found.

We use *Phi* as our metric of choice as it reflects realistic end-use cases for computationally predicted binding sites; such as intensive interrogation using site-directed mutagenesis followed by gel-shift and reporter gene assays. *Phi*-based accuracy combines both the likelihood that a predicted binding site belongs to the set of *bona fide* binding sites (PPV), and the degree to which true binding sites for a given transcription factor have been represented in the computational prediction (sensitivity).

The key disadvantage of the *Phi* is score is that it is undefined when a program fails to return any motif. In this study, we remove such instances when computing average *Phi*, and define *Phi* to be 0 in such cases for the purposes of counting wins and clear wins.

<u>c. Criteria for performance comparison</u>

Ten other motif finding programs were run on the same dataset to provide a point of comparison for SCOPE's performance. These motif finders were chosen because of their popularity and availability in a web server format, and were intended to represent a diverse range of motif models (including consensus, *k*-mismatch and PWM) and search algorithms (including exhaustive, Gibbs sampling, expectation maximization, and several specialized search techniques). Of the programs, one (YMF) is specifically aimed at *S. cerevisiae*, two (BioProspector and MITRA) are aimed at the long, highly degenerate motifs common to prokaryotes, and the others are generalized motif finders.

All programs were run directly from their web site using default parameter settings. The only parameter we specified was species designation, where that option was available. (We were unable to assess the performance of Weeder on prokaryotes because Weeder does not supply a background model for these species).

YMF, whose web server was not available at the time of this study, was run as a downloadable executable. This program is notable in that it uses a type of ensemble method in that the downloadable script runs YMF numerous times under different parameter settings (looking for different lengths of motifs with different ranges of gap lengths) and returns the motifs with the highest scores. Unlike SCOPE, the different motifs are not weighted to make the results comparable and the search remains highly restricted (only six IUPAC bases are explored and motif lengths are capped at eight bases, excluding a possible gap sequence).

d. Statistical analysis of performance comparison results

Over 78 regulons, SCOPE scored in the top three of all eleven motif finders for 63% of the regulons, and in the bottom three of motif finders for 9% of the regulons (Supplementary Figure 1). SCOPE's nearest competitor by each of the criteria was a different program. For instance, Weeder had the second highest average score, (0.18 vs. 0.24 for SCOPE), MEME performed best against SCOPE by the 'clear win' criterion, (losing to SCOPE 31-13), wConsensus had the second highest number of wins (13 vs. 19 for SCOPE), and YMF had the second most scores over 0.50 (7 vs. 8 for SCOPE).

A consistent finding in previous performance comparisons was that all programs perform roughly the same, with no statistically significant differences. While Tompa *et al.* [15] found that Weeder slightly outperformed the other programs and Sinha and Tompa [10] found that YMF outperformed AlignAce and MEME, neither result was statistically significant. Likewise, Hu and colleagues [14] found no clear differences in their performance comparison on prokaryotes and Harbison *et al.* [16] found no clear superiority among the 6 motif finders they ran on sequences identified by ChIP (Chromatin Immunoprecipitation) experiments for 172 transcription factors from *S. cerevisiae*. Consistent with these results, we found no differences between the ten other motif finding programs in this performance comparison (assessed using a one way ANOVA, p = 0.13).

In contrast, when SCOPE was added to the comparison, we found a statistically significant difference between all eleven programs (as assessed by a one-way ANOVA, p=0.00016). The accuracy score of all motif finders was found to be dependent on the identity of the regulon (as assessed by a two-way ANOVA without replication, p=$1.81\text{x}10^{-13}$ for regulon).

We assessed the statistical significance of SCOPE's performance margin using permutation testing, a standard nonparametric statistical technique. The use of permutation testing was particularly appropriate, given the dependency of the accuracy score on the identity of the regulon, since this technique preserves the underlying structure of the data. We randomly permuted each row of Supplementary Table 2 (shuffling motif finder scores for a given regulon) and recomputed the averages for each column (each motif finder). We then computed average accuracies and the difference between the top two averages for 50,000 iterations (the cumulative distribution function for this statistic is shown in Supplementary Figure 2). The difference in accuracy between SCOPE and Weeder (the second-highest scoring program) was 0.065. For a difference of this magnitude on the dataset in question, the p-value from the permutation test was $2 \text{ x } 10^{-5}$. In comparison, without SCOPE, the permutation test p-value was 0.44 for the difference

in accuracy between the next two motif finders (Weeder and RSAT, which differed by 0.008). Taken together with results of the single factor ANOVAs (with and without SCOPE), this analysis suggests that SCOPE maintains a unique, statistically significant performance margin over the other motif finders run on this dataset, under the specific conditions chosen for this test, and on the dataset in question.

Breaking down the performance comparison by species, SCOPE outperformed the other motif finders in 38 out of the 40 categories arising from combinations of species and motif finder. The only exceptions were *E. coli*, where AlignACE (with an average accuracy of 0.18) outperformed SCOPE (0.16), and *D. melanogaster*, where RSAT (with an average accuracy of 0.09) outperformed SCOPE (0.08) (Supplementary Figure 3). To reduce the dependence of our final result on the species mix, we also investigated the effect of weighting all four species equally in computing the final accuracy. Using this weighted average, SCOPE (average accuracy of 0.19) outperformed the next highest motif finder, YMF (average accuracy of 0.14).

Figures and Tables

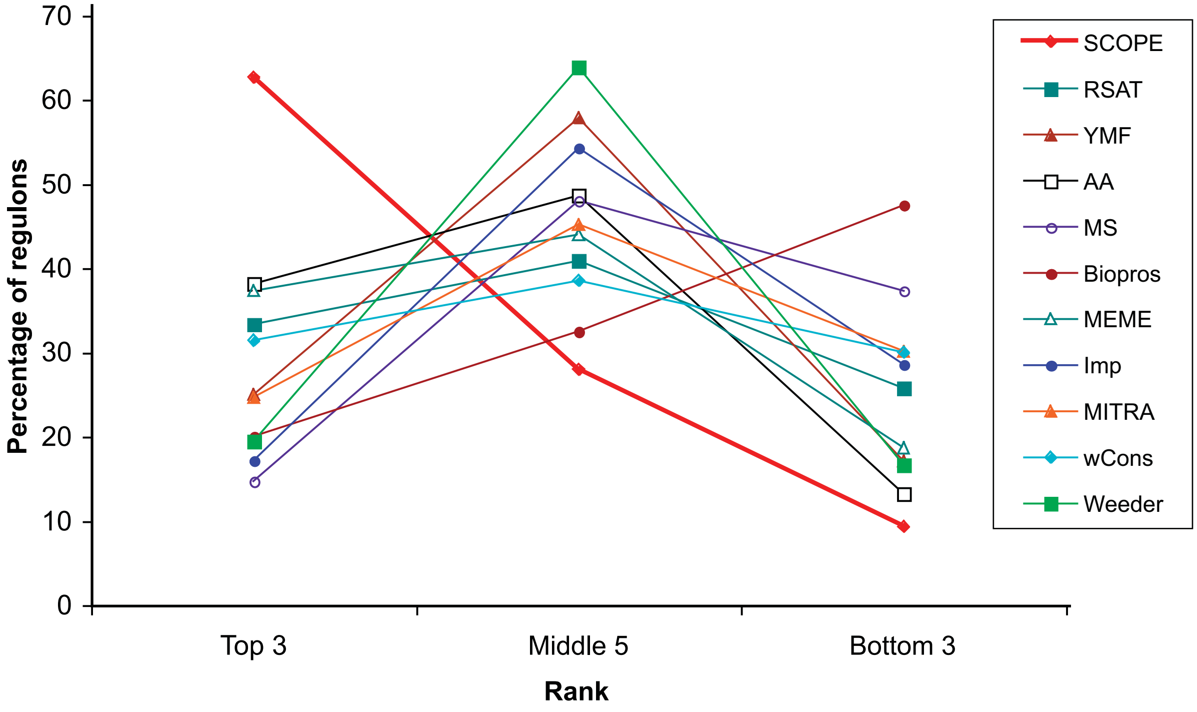

Supplementary Figure 1: Performance according to relative rank. For each regulon, the programs were ranked according to their accuracy. This plot shows the percentage of regulons for which a given program's accuracy places it in the Top 3, Middle 5 or Bottom 3 relative to the other 10 programs. Thus, for 63% of the regulons, SCOPE was among the best three programs, while it was among the worst three programs for 9% of the regulons. None of the other programs consistently rank in the top 3 relative to the other programs, consistent with our finding that the 10 other programs perform similarly to each other. Program abbreviations and references are defined in Table 1 in the main paper.

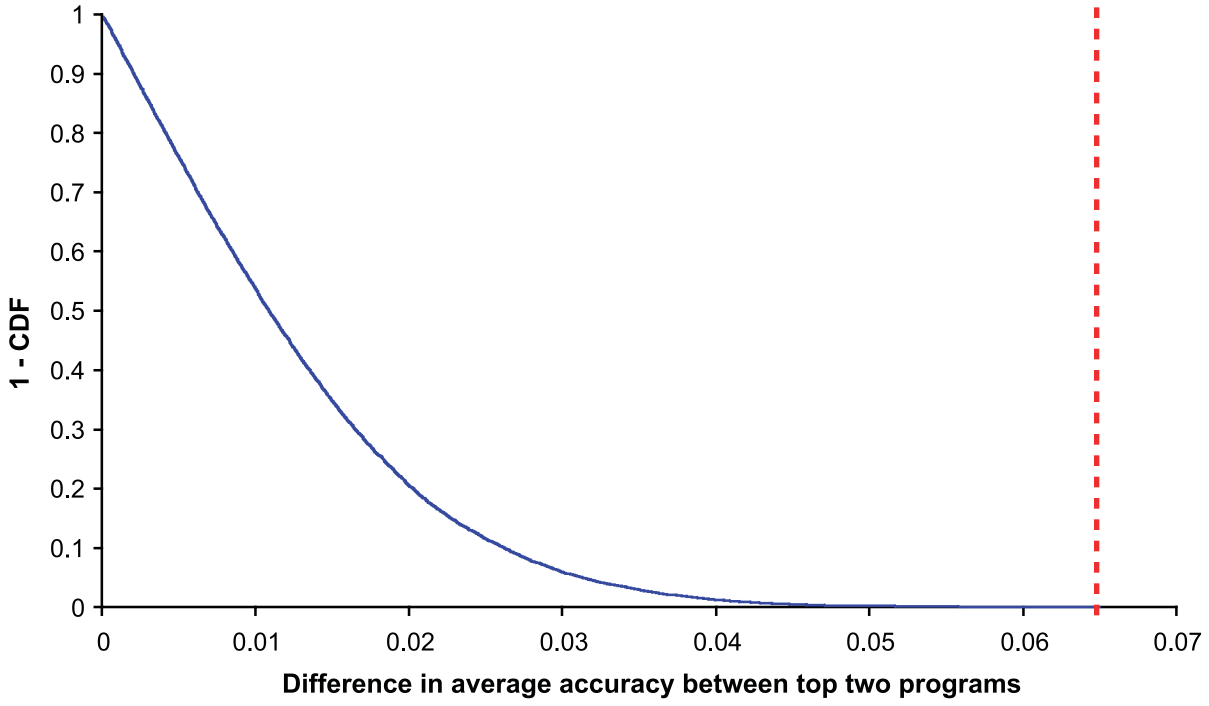

Supplementary Figure 2: Distribution of difference in average accuracy between the top two programs over 50,000 iterations of a permutation test. For each iteration, we independently permuted each row of Supplementary Table 2, recomputed the averages for each column (program), and measured the difference in accuracy between the top two programs for that iteration. Red line indicates the actual difference between SCOPE and Weeder in the real data set ($p=2 \times 10^{-5}$).

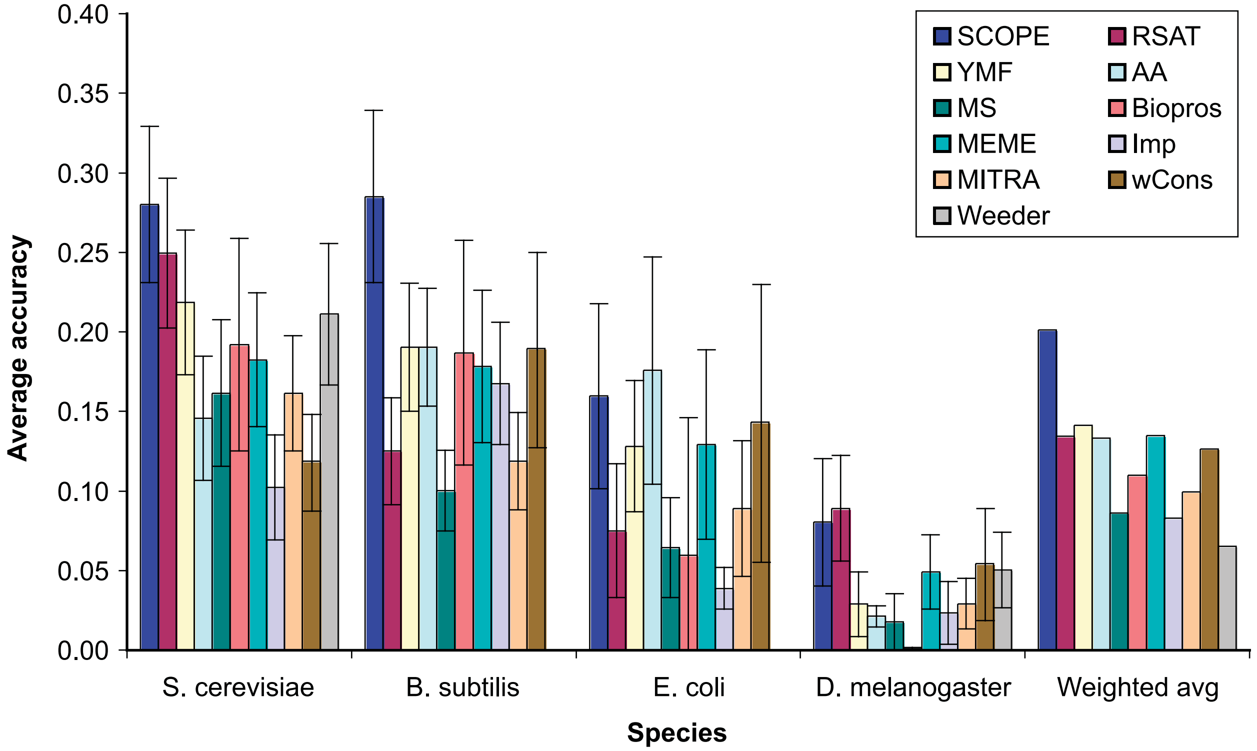

Supplementary Figure 3: Breakdown of performance by organism. *Weighted avg* is the average of each of the organism averages (each organism is weighted at 25%). Program abbreviations and citations are defined in Table 1 in the main paper. Bars represent standard error.

Species	Regulon	SCOPE	RSAT	YMF	AA	MS	BioP	MEME	Imp	MITRA	wCons	Weeder
S. cerevisiae	ABF1	0.29	0.35	0.02	0.29	0.00	0.04	0.00	0.01	0.02	0.03	0.03
	BAS1	0.35	0.36	0.36	0.00	0.00	0.04	0.00	0.00	0.00	0.00	0.20
	CPF1	0.62	-	0.44	0.00	0.54	-	0.62	0.07	0.48	0.00	0.44
	CSRE	0.33	0.25	0.07	0.00	0.10	-	0.49	0.04	0.23	0.00	0.09
	GAL4	0.20	0.02	0.16	0.18	0.12	0.11	0.13	0.10	0.12	0.11	0.10
	GATA	0.49	0.73	0.56	0.24	0.22	0.43	0.14	0.25	0.23	0.09	0.36
	GCN4	0.28	0.29	0.29	0.02	0.26	0.28	0.00	0.25	0.01	0.03	0.50
	GCR1	0.02	0.24	0.10	0.04	0.05	0.10	0.08	0.03	0.25	0.00	0.07
	GLN3	0.39	0.00	0.00	0.02	0.79	-	0.00	0.00	0.05	0.00	0.00
	HAP1	0.05	0.20	0.06	0.14	0.10	0.00	0.22	0.10	0.00	0.03	0.06
	HAP2	0.00	-	0.00	0.02	0.00	-	0.18	0.01	0.00	0.25	0.01
	HSE1	0.44	0.38	0.38	0.13	0.43	0.05	0.29	0.05	0.16	0.19	0.37
	MATA1	0.19	0.10	0.13	0.26	0.17	-	0.49	0.00	0.11	0.14	0.06
	MATA2	0.40	0.28	0.23	0.08	0.15	0.04	0.41	0.00	0.06	0.54	0.34
	MCB	0.62	0.64	0.64	0.02	0.46	0.49	0.06	0.46	0.34	0.28	0.50
	MCM1	0.41	0.21	0.17	0.53	0.07	0.16	0.33	0.19	0.07	0.45	0.12
	MIG1	0.11	0.22	0.29	0.02	0.14	0.02	0.16	0.04	0.10	0.05	0.15
	PDR3	0.70	0.46	0.74	0.51	0.76	0.75	0.42	0.44	0.41	0.01	0.82
	PHO2	0.00	0.00	0.00	0.06	0.08	-	0.10	0.01	0.01	0.13	0.07
	PHO4	0.25	0.21	0.18	0.24	0.23	-	0.21	0.19	0.33	0.25	0.14
	RAP1	0.19	0.18	0.18	0.26	0.02	0.00	0.02	0.19	0.01	0.00	0.24
	REB1	0.44	0.36	0.36	0.00	0.01	0.46	0.27	0.03	0.27	0.00	0.36
	ROX1	0.07	0.33	0.18	0.05	0.05	-	0.09	0.00	0.47	0.00	0.15
	RPA	0.25	0.00	0.00	0.00	0.00	-	0.08	0.00	0.00	0.00	0.00
	SCB	0.77	0.52	0.50	0.55	0.07	-	0.09	0.00	0.42	0.22	0.49
	SFF	0.00	0.00	0.00	0.10	0.00	-	0.00	0.00	0.00	0.23	0.00
	STE12	0.11	0.65	0.50	0.08	0.24	0.00	0.00	0.00	0.49	0.00	0.34
	TBP	0.00	0.00	0.01	0.03	0.00	0.19	0.02	0.00	0.02	0.00	0.00
	UASCAR	0.08	0.04	0.02	0.11	0.00	-	0.10	0.00	0.05	0.22	0.02
	UASPHR	0.03	0.06	0.03	0.04	0.11	0.00	0.03	0.13	0.07	0.05	0.10
	UIS	0.24	0.00	0.01	0.03	0.00	-	0.09	0.07	0.03	0.23	0.09
	URS1H	0.67	0.41	0.40	0.65	0.00	0.49	0.73	0.60	0.38	0.28	0.54
B. subtilis	AbrB	0.15	0.10	0.15	0.14	0.14	0.00	0.07	0.05	0.20	0.03	N.A.
	AhrC	0.31	0.00	0.17	0.18	0.00	0.25	0.08	0.24	0.14	0.00	N.A.
	AraR	0.44	0.23	0.28	0.41	0.31	0.35	0.15	0.23	0.49	0.00	N.A.
	CcpA	0.41	0.10	0.24	0.38	0.29	0.34	0.47	0.57	0.34	0.60	N.A.
	CcpC	0.39	0.00	0.22	0.05	0.16	-	0.00	0.08	0.00	0.05	N.A.
	comA	0.27	0.19	0.19	0.17	0.00	-	0.46	0.19	0.05	0.00	N.A.
	comK	0.22	0.00	0.12	0.14	0.07	0.00	0.04	0.11	0.19	0.09	N.A.
	degU	0.05	0.00	0.03	0.07	0.04	0.04	0.08	0.01	0.04	0.03	N.A.
	Fnr	1.00	0.27	0.39	0.00	0.28	0.64	0.53	0.39	0.07	0.81	N.A.
	GerE	0.14	-	0.06	0.14	0.13	-	0.19	0.05	0.15	0.00	N.A.
	GlnR	0.12	-	0.00	0.10	0.00	-	0.00	0.24	0.00	0.00	N.A.
	GltC	0.36	-	0.28	0.32	0.17	-	0.32	0.00	0.00	0.00	N.A.
	Hpr	0.30	0.04	0.05	0.17	0.07	-	0.00	0.04	0.04	0.00	N.A.
	LexA	0.61	0.24	0.58	0.64	0.00	-	0.60	0.26	0.00	0.55	N.A.
	LicT	0.16	0.08	0.10	0.09	0.15	-	0.20	0.18	0.05	0.19	N.A.
	Mta	0.17	0.11	0.09	0.12	0.00	-	0.09	0.20	0.14	0.45	N.A.
	PhoB	0.13	-	0.07	0.06	0.06	0.11	0.05	0.04	0.06	0.06	N.A.
	PurR	0.21	0.00	0.15	0.09	0.12	0.11	0.08	0.09	0.00	0.21	N.A.
	ResD	0.31	0.11	0.11	0.05	0.00	-	0.00	0.02	0.09	0.02	N.A.
	SigD	0.15	0.07	0.08	0.15	0.00	-	0.03	0.04	0.21	0.08	N.A.
	SigW	0.21	0.15	0.15	0.21	0.01	0.20	0.60	0.31	0.32	0.27	N.A.
	SigX	0.13	0.08	0.11	0.07	0.05	0.15	0.25	0.09	0.05	0.08	N.A.
	Spo0A	0.47	0.47	0.62	0.42	0.23	0.42	0.00	0.58	0.27	0.36	N.A.
	SpoIIID	0.04	0.19	0.06	0.17	0.08	0.00	0.14	0.08	0.10	0.04	N.A.
	TnrA	0.00	0.00	0.18	0.21	0.27	0.02	0.18	0.20	0.08	0.22	N.A.
	XylR	0.67	0.33	0.49	0.43		-	0.02	0.07	0.00	0.79	N.A.
E. coli	ArcA	0.05	0.00	0.01	0.00	0.00	-	0.00	0.04	0.07	0.00	N.A.
	CaiF	0.28	0.17	0.19	0.24	0.20	-	0.21	0.03	0.32	0.47	N.A.
	Crp	0.21	0.01	0.10	0.25	0.07	0.03	0.07	0.08	0.12	0.22	N.A.
	DnaA	0.02	-	0.14	0.09	0.00	-	0.32	0.00	0.09	0.00	N.A.
	Fis	0.05	0.00	0.05	0.05	0.01	0.01	0.04	0.04	0.05	0.02	N.A.
	Fnr	0.41	0.19	0.29	0.52	0.10	0.00	0.32	0.07	0.01	0.41	N.A.
	GlpR	0.10	0.09	0.20	0.23	0.14	-	0.23	0.08	0.06	0.31	N.A.
	IHF	0.10	0.02	0.03	0.02	0.03	0.01		0.02	0.03	0.01	N.A.
	Lpr	0.13	-	0.09	0.11	0.00	-	0.00	0.02	0.00	0.00	N.A.
	narL	0.26	0.11	0.20	0.25	0.09	0.25	0.11	0.01	0.15	0.00	N.A.
D. melanogaster	Bcd	0.14	0.14	0.09	0.00	0.00	0.00	0.04	0.02	0.03	0.08	0.05
	Cad	0.02	0.05	0.00	0.03	0.00	0.00	0.06	0.00	0.01	0.00	0.02
	En	0.01	0.10	0.01	0.03	0.00	-	0.10	0.00	0.05	0.00	0.06
	Ftz-F1	0.00	0.00	0.00	0.02	0.00	-	0.15	0.00	0.00	0.17	0.04
	Hb	0.27	0.23	0.01	0.01	0.00	0.00	0.04	-	0.08	0.03	-
	Kni	0.07	0.00	0.00	0.01	0.00	0.00	0.00	0.01	0.01	0.03	0.00
	Kr	0.08	0.10	0.12	0.01	0.02	0.00	0.02	0.00	0.01	0.03	0.17
	Pho	0.17	0.08	0.00	0.03	0.00	0.00	0.10	0.00	0.08	0.00	0.04
	PRD(HD)	0.01	0.05	0.00	0.04	0.03	-	0.00	0.11	-	0.00	0.02
	Ttk	0.04	0.15	0.05	0.04	0.12	-	0.00	0.08	0.00	0.21	0.05

Supplementary Table 1: Accuracy of each program on each regulon. Accuracy was measured using the *Phi* score of the reported binding sites against the known binding sites. – indicates the program failed to return any result. In some cases (e.g. Bioprospector), missing data is reported for small regulons because the program will not run datasets under a certain size. In other cases (e.g. RSAT) missing data reflects the program's assertion that no significant results exist. Finally, some missing data resulted from the failure of the program to return any results or error messages in a reasonable time frame (measured in days). All analyses ignored missing data, with the exception of Sensitivity, for which failure to predict any motifs is equivalent to reporting wrong motifs. N.A. indicates Weeder did not support this organism. No summary statistics for Weeder were computed using these regulons.

	SCOPE	RSAT	YMF	AA	MS	Biopros	MEME	Imp	MITRA	wCons	Weeder
Average	0.24	0.17	0.17	0.15	0.11	0.15	0.16	0.11	0.12	0.14	0.18
Stderr	0.02	0.02	0.02	0.02	0.02	0.03	0.02	0.02	0.02	0.02	0.03
Wins	19	5	3	5	2	2	11	3	6	13	3
scores ≥ 0.50	8	4	7	6	3	2	5	3	0	5	4
scores ≥ 0.33	21	12	14	10	5	9	12	6	9	10	11
scores ≥ 0.20	39	24	22	22	14	12	23	14	18	24	13
Regulons returned	78	70	78	78	78	44	78	77	77	78	41
clear win for SCOPE vs	-	24	24	25	37	18	31	36	36	33	15
clear loss for SCOPE vs	-	8	6	6	3	1	13	5	4	13	3

Supplementary Table 2: Summary results for performance comparisons on all regulons. In situations where a program failed to return any motif for a regulon, the accuracy is undefined and was not used to calculate the averages. A "win" is a regulon for which a program has the highest accuracy, and that accuracy is at least 0.10. A "clear win (loss)" is a regulon for which SCOPE's accuracy was at least 0.10 higher (lower) than the other program. Abbreviations and citations are defined in Table 1 in the main paper.

References

1. Carlson JM, Chakravarty A, Gross RH: **BEAM: a beam search algorithm for the identification of cis-regulatory elements in groups of genes.** *J Comput Biol* 2006, **13:**686-701.
2. Carlson JM, Chakravarty A, Khetani RS, Gross RH: **Bounded search for de novo identification of degenerate cis-regulatory elements.** *BMC Bioinformatics* 2006, **7:**254.
3. Chakravarty A, Carlson JM, Khetani RS, DeZiel CE, Gross RH: **SPACER: Identification of cis-regulatory elements with non-contiguous critical residues.** *Bioinformatics* 2007.
4. Russell S, Norvig P: *Artificial Intelligence: A Modern Approach.* Prentice hall; 1995.
5. Sinha S, Tompa M: **A statistical method for finding transcription factor binding sites.** *Proc Int Conf Intell Syst Mol Biol* 2000, **8:**344-354.
6. van Helden J, Andre B, Collado-Vides J: **Extracting regulatory sites from the upstream region of yeast genes by computational analysis of oligonucleotide frequencies.** *J Mol Biol* 1998, **281:**827-842.
7. Blanchette M, Sinha S: **Separating real motifs from their artifacts.** *Bioinformatics* 2001, **17 Suppl 1:**S30-38.
8. Zhu J, Zhang MQ: **SCPD: a promoter database of the yeast Saccharomyces cerevisiae.** *Bioinformatics* 1999, **15:**607-611.
9. Wingender E, Dietze P, Karas H, Knuppel R: **TRANSFAC: a database on transcription factors and their DNA binding sites.** *Nucleic Acids Res* 1996, **24:**238-241.
10. Sinha S, Tompa M: **YMF: A program for discovery of novel transcription factor binding sites by statistical overrepresentation.** *Nucleic Acids Res* 2003, **31:**3586-3588.
11. Munch R, Hiller K, Barg H, Heldt D, Linz S, Wingender E, Jahn D: **PRODORIC: prokaryotic database of gene regulation.** *Nucleic Acids Res* 2003, **31:**266-269.
12. Papatsenko DA, Makeev VJ, Lifanov AP, Regnier M, Nazina AG, Desplan C: **Extraction of functional binding sites from unique regulatory regions: the Drosophila early developmental enhancers.** *Genome Res* 2002, **12:**470-481.
13. Benos PV, Lapedes AS, Stormo GD: **Is there a code for protein-DNA recognition? Probab(ilistical)ly.** *Bioessays* 2002, **24:**466-475.
14. Hu J, Li B, Kihara D: **Limitations and potentials of current motif discovery algorithms.** *Nucleic Acids Res* 2005, **33:**4899-4913.
15. Tompa M, Li N, Bailey TL, Church GM, De Moor B, Eskin E, Favorov AV, Frith MC, Fu Y, Kent WJ, et al: **Assessing computational tools for the discovery of transcription factor binding sites.** *Nat Biotechnol* 2005, **23:**137-144.
16. Harbison CT, Gordon DB, Lee TI, Rinaldi NJ, Macisaac KD, Danford TW, Hannett NM, Tagne JB, Reynolds DB, Yoo J, et al: **Transcriptional regulatory code of a eukaryotic genome.** *Nature* 2004, **431:**99-104.
